# Malignant Transformation of Recurrent Synovial Chondromatosis: A Case Report and Review

**DOI:** 10.7759/cureus.5839

**Published:** 2019-10-04

**Authors:** John W Urwin, Kumarasen Cooper, Ronnie Sebro

**Affiliations:** 1 Medicine, Beth Israel Deaconess Medical Center / Harvard Medical School, Boston, USA; 2 Pathology, Hospital of the University of Pennsylvania, Philadelphia, USA; 3 Radiology, Perelman School of Medicine at the University of Pennsylvania, Philadelphia, USA

**Keywords:** synovial chondromatosis, malignant degeneration

## Abstract

Chondrosarcoma is the second most common primary malignant bone tumor. While the majority arrive de novo, a minority arise from malignant transformation of benign neoplasms, such as osteochondromas. Rarely, chondrosarcomas have been found to originate from other preexisting lesions, such as synovial chondromatosis.

A 44-year-old male with a history of a spinal osteochondroma presented with one year of left hip pain and decreased range of motion. On examination, he had a palpable, irregular fullness in the left groin that was minimally tender to palpation. Radiographs and CT of the hip showed extensive soft tissue calcifications and erosion of the femoral neck. The lesion was debulked surgically and histologically diagnosed as synovial osteochondromatosis with no evidence of atypia or cellularity.

One year later, his residual disease progressed and resulted in increasingly limited range of motion. He underwent left total hip arthroplasty with simultaneous debulking and the lesion was once again diagnosed as synovial osteochondromatosis.

Two months postoperatively, the patient developed a new focus of calcification around the hip joint that was thought to be recurrent disease. Six months later, due to worsening symptoms, he underwent a repeat CT scan. This scan demonstrated extensive intra-articular disease extending into the iliopsoas bursa and around total hip arthroplasty, as well as a new soft tissue nodule with foci of calcification in the left gluteus maximus. The new lesion was debulked surgically and diagnosed as a grade 1 chondrosarcoma.

Chondrosarcoma arising from synovial chondromatosis is a rare presentation of the second most common primary malignant bone tumor. It typically presents as an indolent, slowly growing painful mass of large joints in middle aged men. Conventional radiography shows punctate opacities, while MRI and CT reveal diffuse soft tissue calcification and cortical erosion. Low-grade chondrosarcomas are treated with intralesional curettage and adjuvant therapy, while higher grade chondrosarcomas are treated with wide, en bloc excision. Malignant transformation should be considered in any patient presenting with worsening symptoms and a history of a benign bony lesion.

## Introduction

Synovial chondromatosis was first described by Ambrose Pare in 1558 and Laennic in 1813 [1,2]. The exact prevalence of synovial chondromatosis is unknown [3]. It is characterized by metaplasia of synovial connective tissue into cartilaginous or osteochondral bodies [4]. These foci can be either intra-articular or extra-articular in bursa or tendon sheaths [4]. Synovial chondromatosis can arise either in non-oseoarthritis joints as primary synovial chondromatosis or in osteoarthritic joints as secondary synovial chondromatosis. Primary synovial chondromatosis usually affects individuals in the third to fifth decades of life, with men being affected two to four times more frequently than women [3]. Secondary synovial chondromatosis is more common than primary synovial chondromatosis and presents later in life, typically during the fifth or sixth decade of life [4].

The exact etiology of synovial chondromatosis is unknown. The knee is the most commonly involved joint followed by hip, shoulder, elbow, ankle, and wrist, but it has been reported in over 30 different locations [3,4]. Synovial chondromatosis has been associated with a variety of genetic abnormalities, including aberrations in chromosome, TGFB, and the hedgehog signaling pathways [5-7].

Primary synovial chondromatosis typically presents indolently as a slow-growing intra-articular masses of large joints. It can cause joint pain, decreased range of motion, and joint stiffness, and often the diagnosis is suggested from radiographs [8]. Primary synovial chondromatosis can lead to erosive changes of the joint and secondary osteoarthritis of the joint [9].

We present a case of synovial chondromatosis with subsequent malignant degeneration into a grade 1 chondrosarcoma of the hip in a 44-year-old male patient with hip pain.

## Case presentation

**Figure 1:**
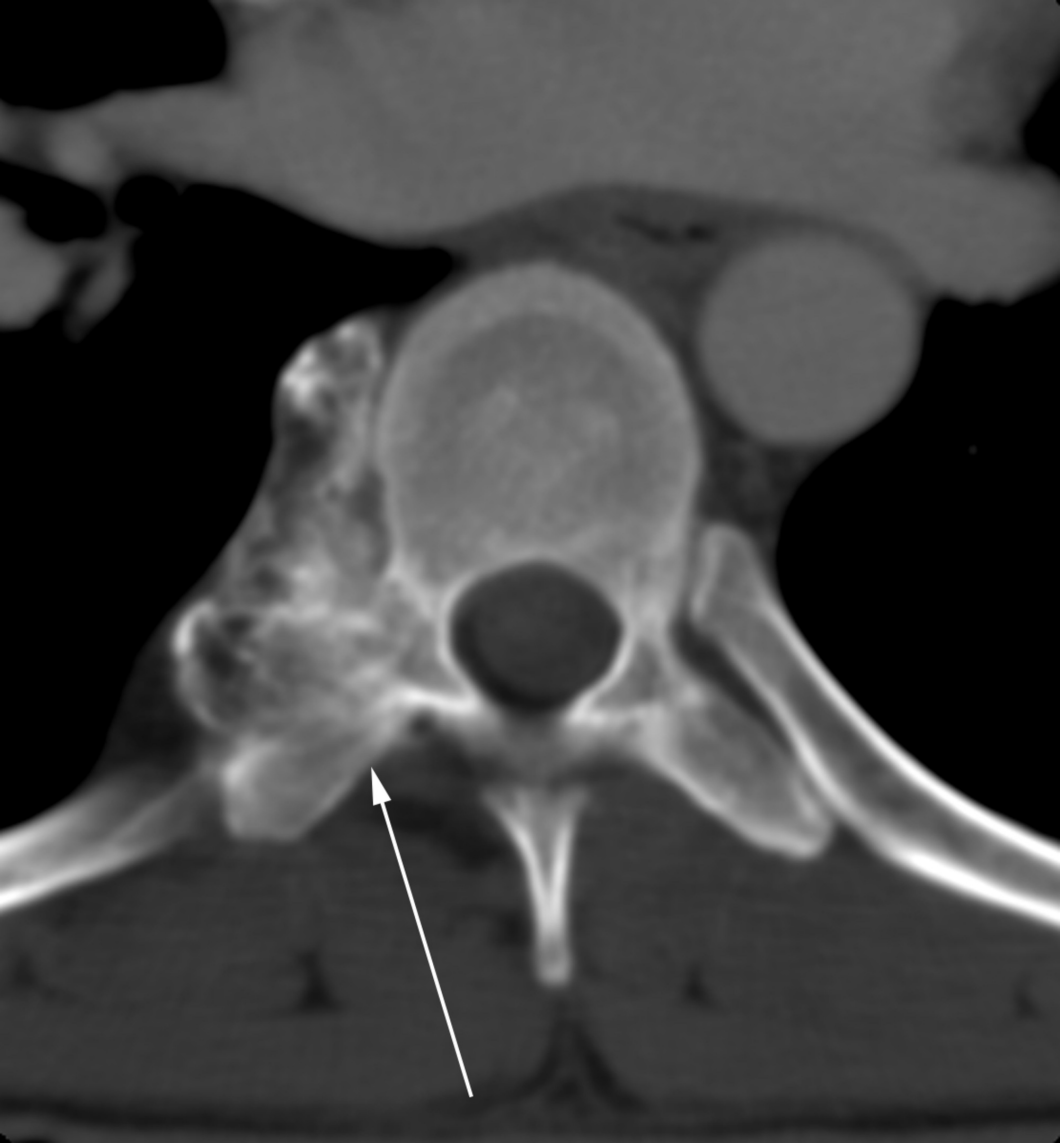
Axial CT image of histologically confirmed osteochondroma of the thoracic spine. White arrow points to primary lesion.

**Figure 2:**
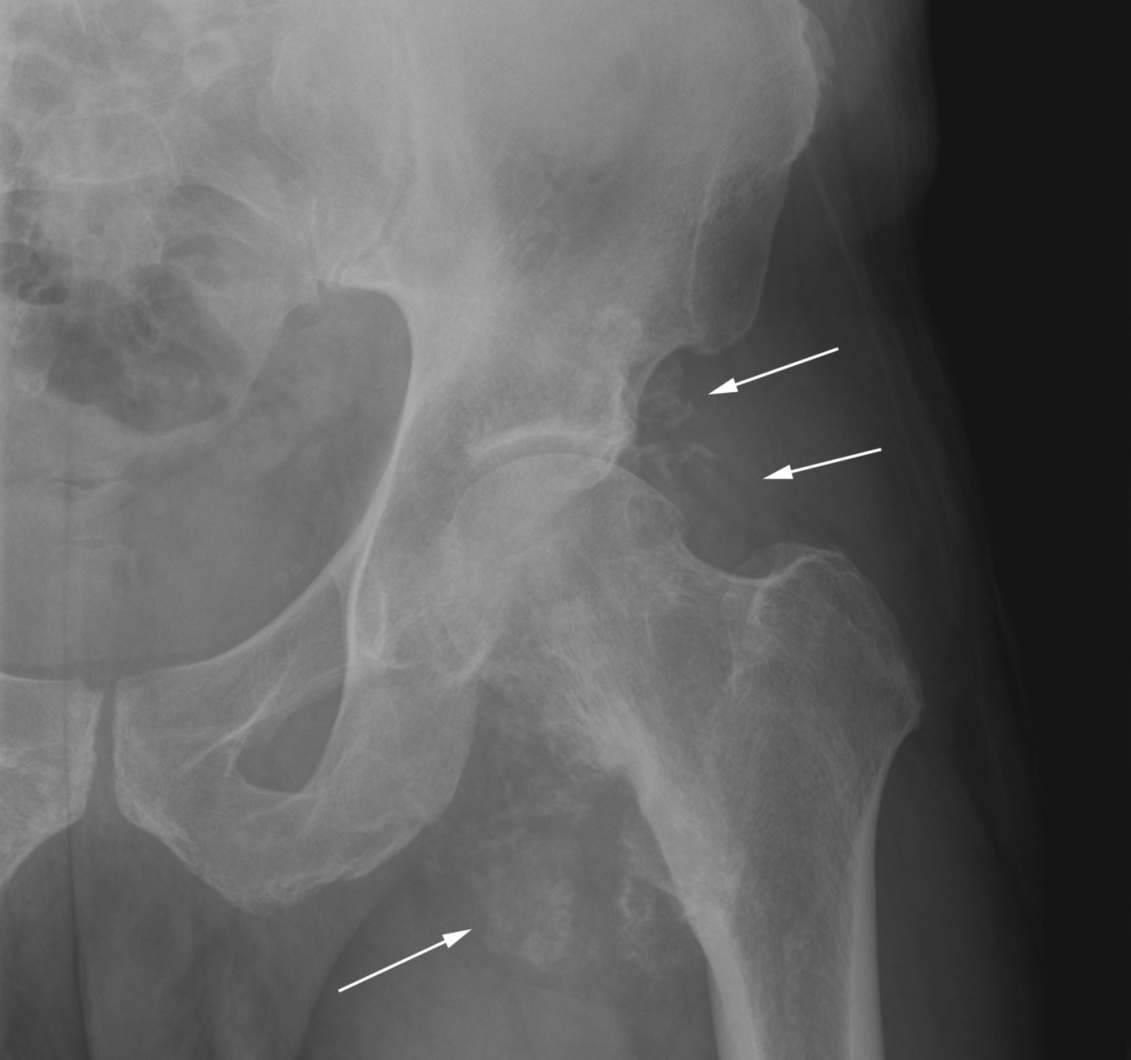
Anterior-posterior radiograph of the left hip. White arrows point to extensive soft tissue calcifications surrounding the hip joint associated with erosion of the femoral neck and mild degenerative changes.

**Figure 3:**
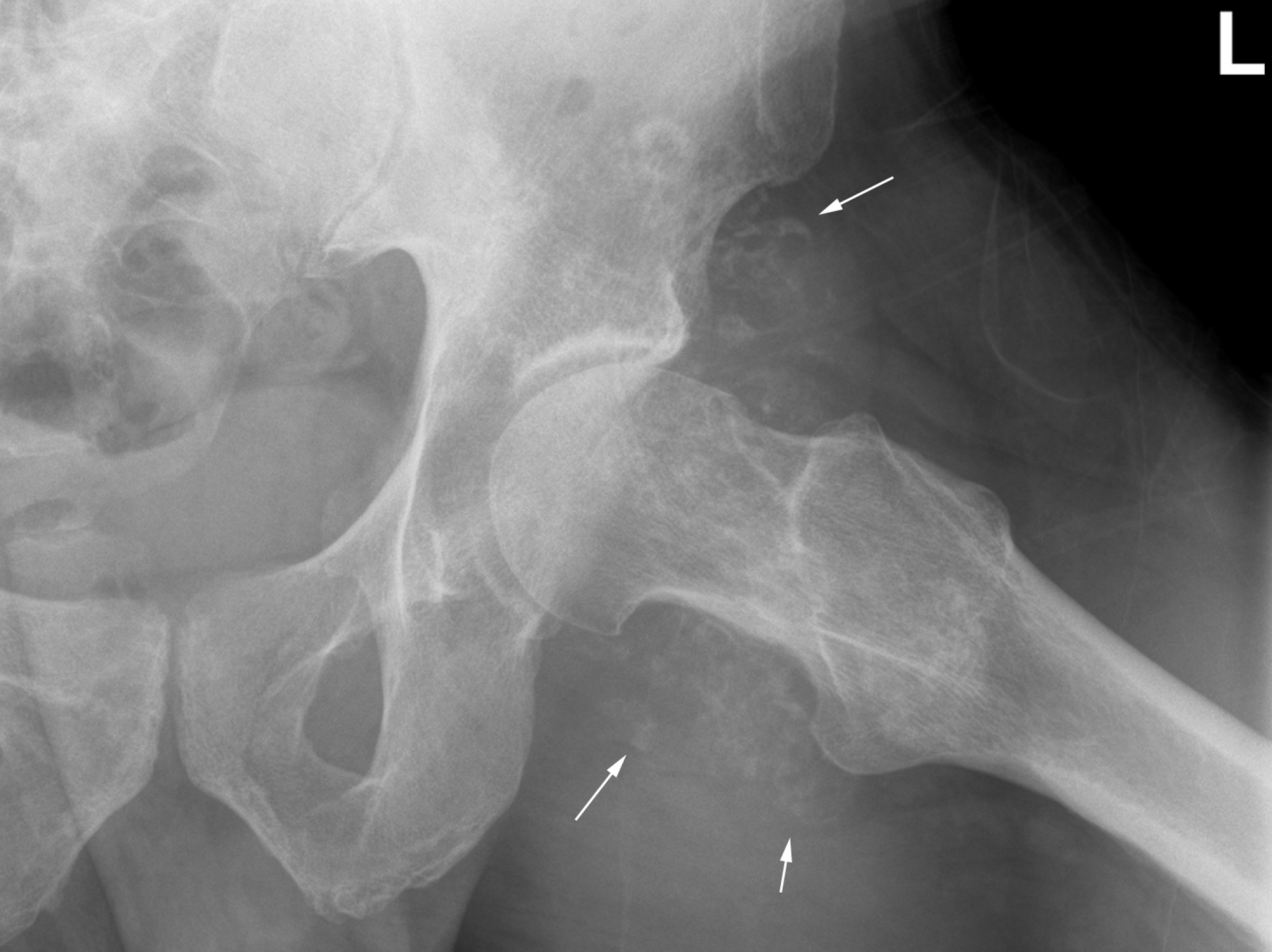
Frog-lateral view of the left hip. White arrows point to extensive soft tissue calcifications surrounding the hip joint associated with erosion of the femoral neck and mild degenerative changes.

A 44-year-old male with history of a biopsy proven spinal osteochondroma (Figure 1) presented with a one-year history of left hip pain and a slowly enlarging left hip mass. He reported an indolent onset of left hip discomfort with progressive loss of range of motion over the year. He walked unassisted and took no medication for pain at the time of presentation. His pain was localized primarily to the left groin, and was worst with flexion/extension of the left hip. He reported occasional left anterior thigh burning pain that is worst with prolonged sitting. He denied radiation of pain down past the calf or weakness of the leg or foot. He presented with a firm prominence along the lateral groin. His physical examination showed a palpable, irregular fullness in the left groin that was minimally tender to palpation. Hip flexion was to 80° but limited by pain. He had hip extension to the neutral position. There was no inguinal adenopathy. His radiographs of the left hip (Figures 2 and 3) showed extensive soft tissue calcifications surrounding the hip joint associated with erosion of the femoral neck and mild left hip degenerative changes. The radiographic differential diagnoses included synovial chondromatosis and synovial chondrosarcoma.

**Figure 4:**
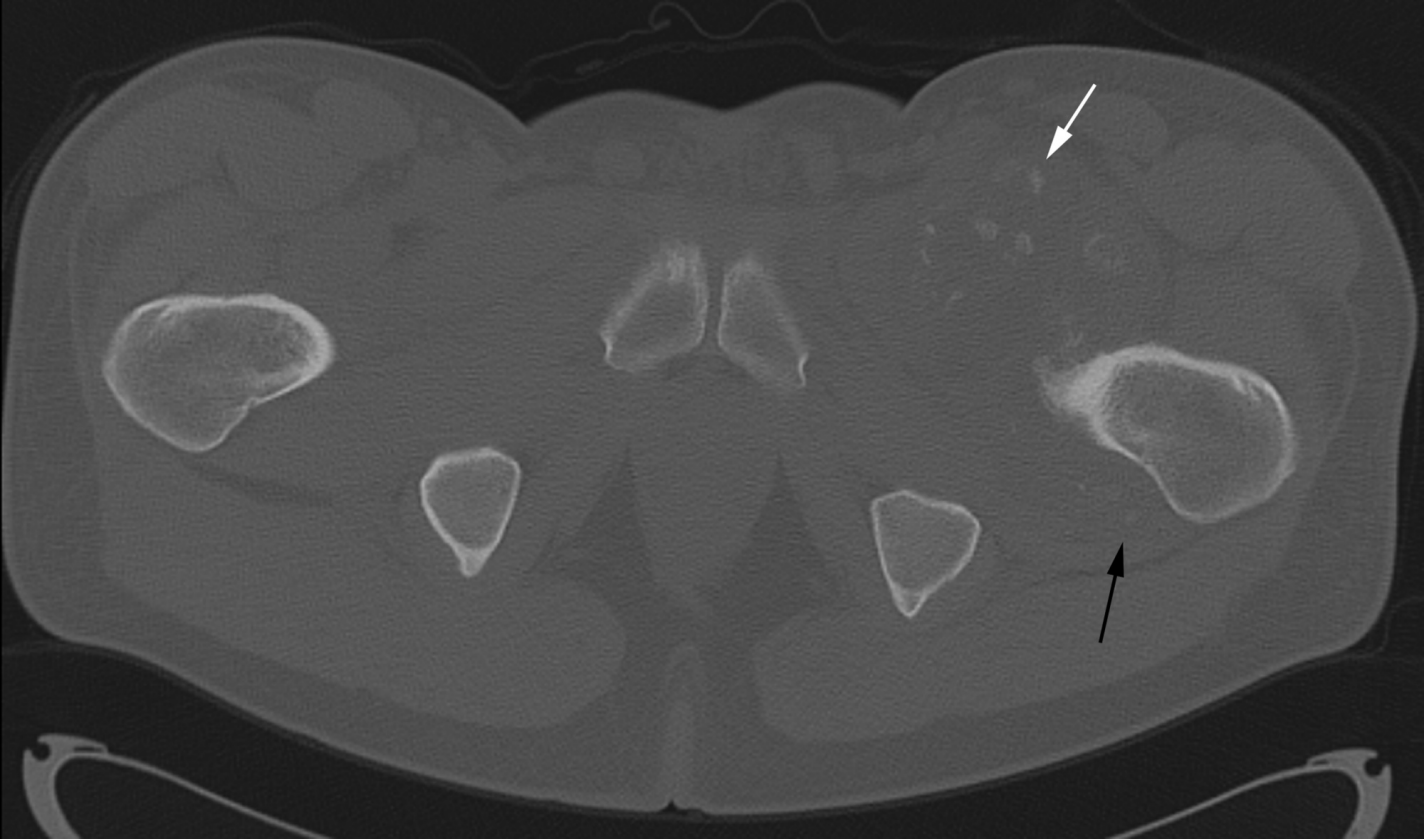
Axial CT of pelvis. White arrow points to anterior intra-articular bodies. Black arrow points to posterior intra-articular bodies.

He subsequently underwent a CT scan, which demonstrated extensive calcifications adjacent to the left hip with asymmetric soft tissue enlargement and mild degenerative changes of the hip joint (Figure 4). The masses were mostly anterior and medial, and the conglomerate measurement was approximately 13 x 7 cm without apparent extension into the pelvis. The differential diagnosis was unchanged, so the patient underwent CT-guided biopsy. He then underwent MRI using a 3T Skyra MRI (Siemens AG, München, Germany) for further evaluation. He underwent open subtotal debulking of his disease because his disease was felt to be too bulky and not amenable to arthroscopy. Both the percutaneous biopsy and final histologic diagnosis from the resected specimens were thought to be most consistent with synovial osteochondromatosis, with no evidence of atypia or cellularity.

**Figure 5:**
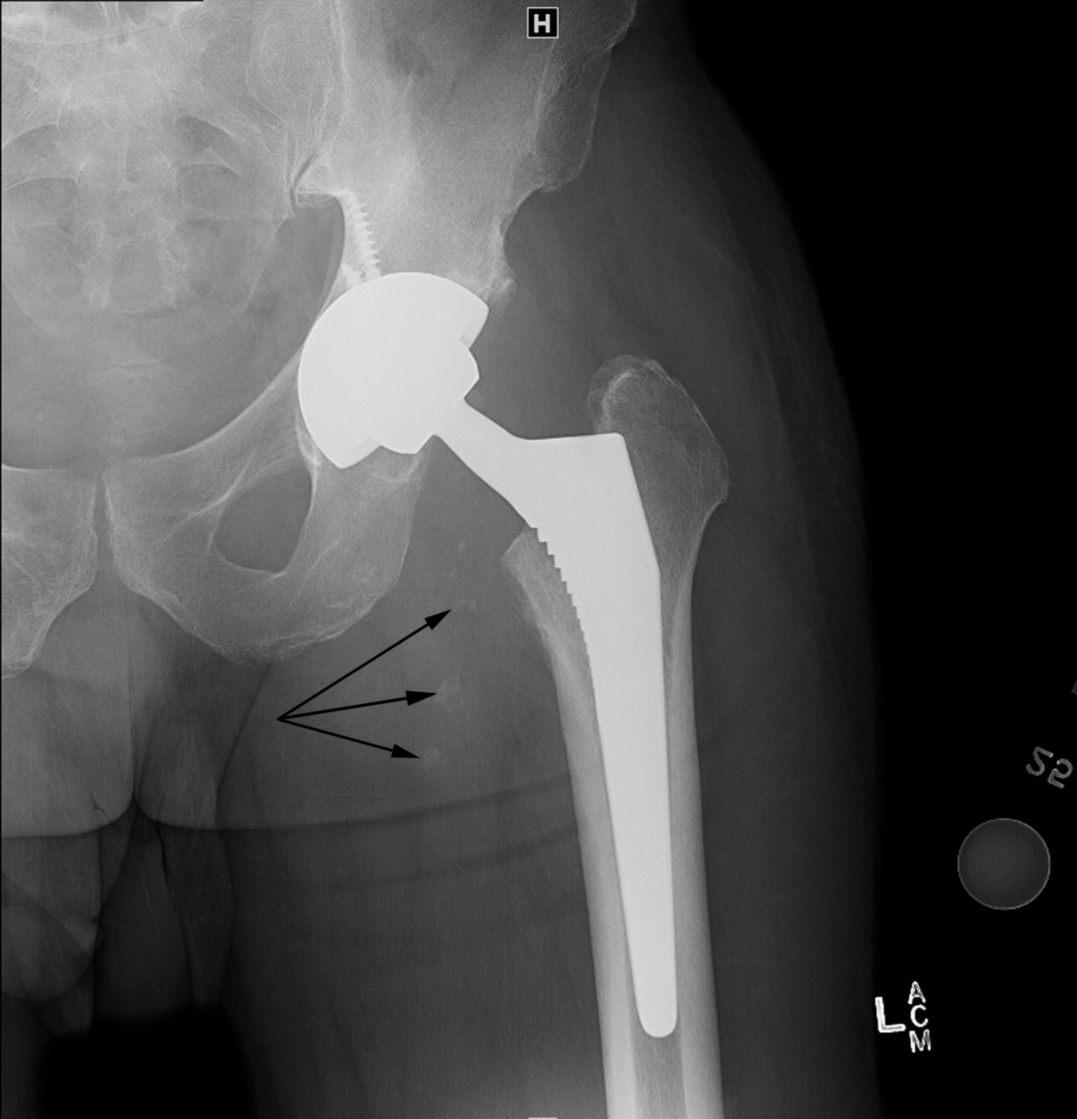
Anterior-posterior radiograph of the left hip two months after left total hip arthroplasty and surgical debulking. Black arrows point to recurrent intra-articular bodies.

**Figure 6:**
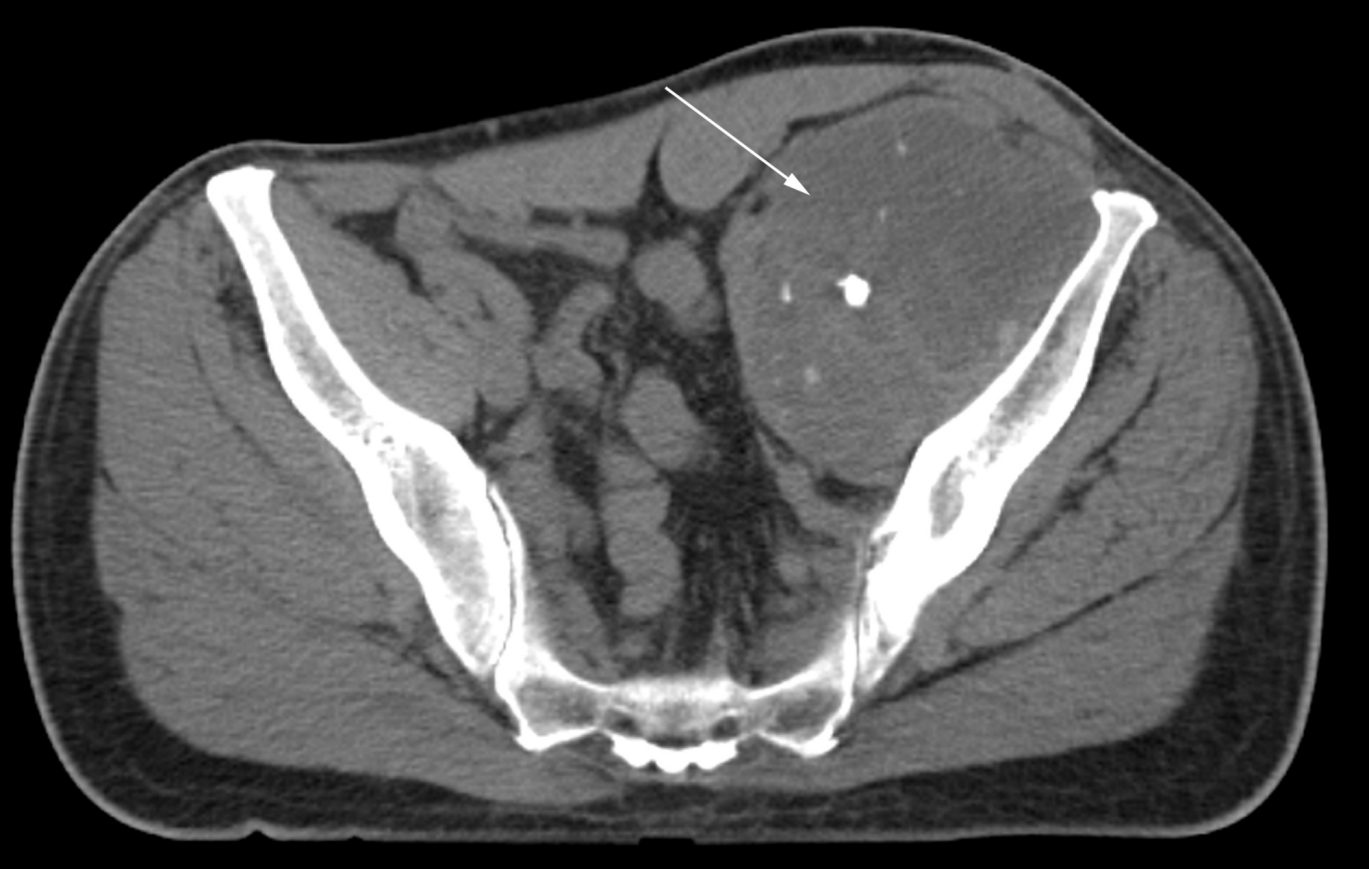
Axial CT of pelvis six months after total hip arthroplasty and surgical debulking. White arrow points to recurrent disease extending into the pelvis in the iliopsoas bursa.

**Figure 7:**
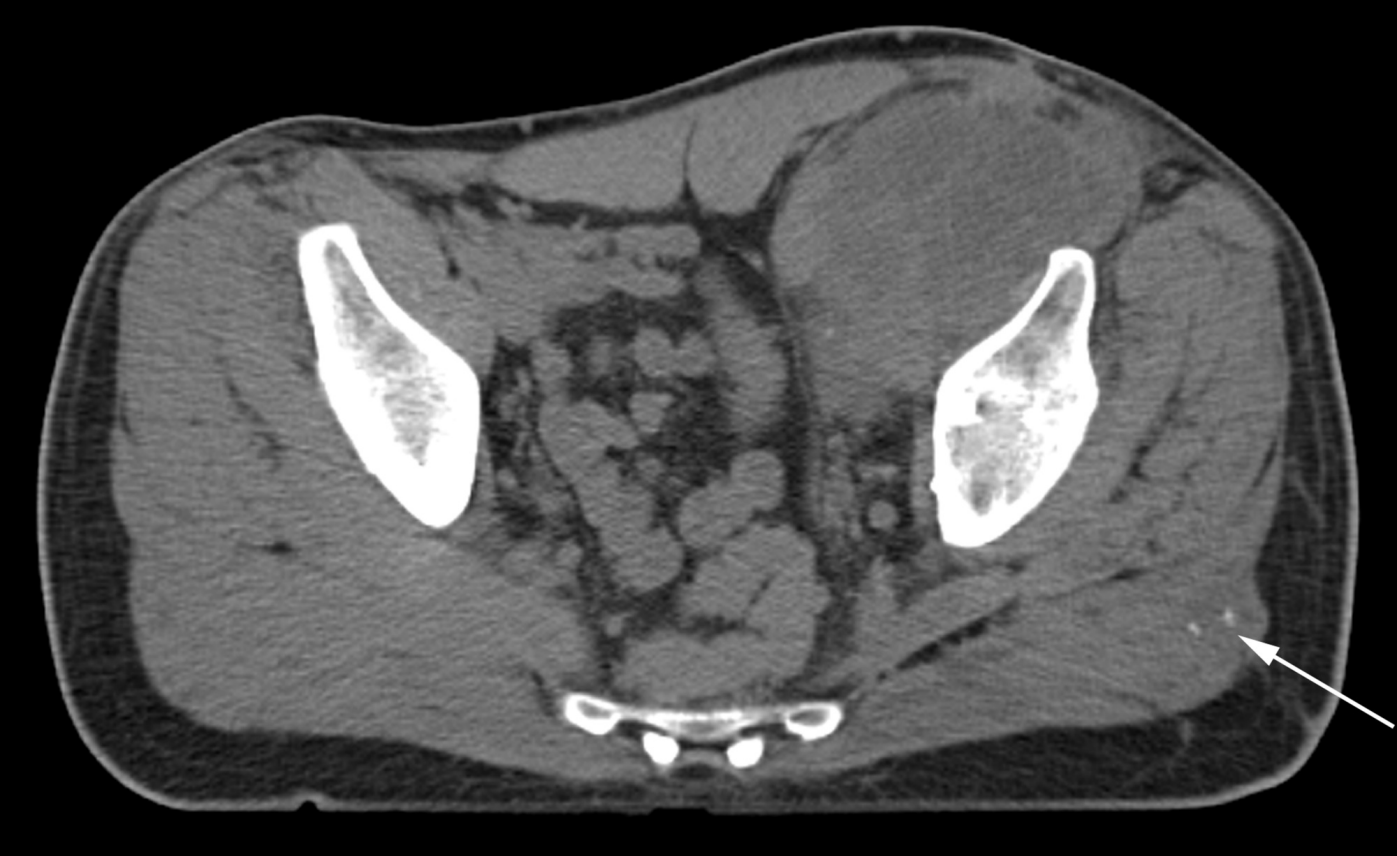
Axial CT of pelvis six months after total hip arthroplasty and surgical debulking. Arrow points to new soft tissue nodule with foci of calcification in the left gluteus maximus.

Approximately one year later, his residual disease progressed and resulted in limiting his range of motion. He underwent a left total hip arthroplasty via a posterior approach with simultaneous debulking of disease. The final histologic diagnosis from the resected femur and intra-articular bodies was synovial osteochondromatosis with erosive change in the femoral head/neck. Approximately two months postoperatively, he developed new foci of calcification around the hip joint on his postoperative radiographic studies (Figure 5), consistent with recurrent disease. Worsening symptoms prompted a repeat CT scan six months later. The CT scan demonstrated extensive intra-articular disease extending into the iliopsoas bursa (Figure 6) and around total hip arthroplasty as well as a new soft tissue nodule with foci of calcification in the left gluteus maximus, consistent with an additional focus of disease (Figure 7).

The patient underwent repeat extensive surgical debulking of his disease. The final histological diagnosis from the excised tumor was grade 1 chondrosarcoma arising from synovial chondromatosis.

## Discussion

Primary synovial chondromatosis is benign, but there are rare case reports of malignant transformation of synovial chondromatosis to a synovial chondrosarcoma. The estimated incidence of synovial chondrosarcoma arising from synovial chondromatosis is approximately 1% to 6% [3]. Chondrosarcoma is a rare malignant bone tumor characterized by their ability to make cartilage [10,11]. It has an incidence of one in 200,000 per year [11]. The majority of chondrosarcomas are de novo primary neoplasms; however, a minority arise from malignant transformation of benign neoplasms, such as osteochondromas and enchondromas, or in this case synovial chondromatosis [3,12]. Bertoni et al. evaluated 10 patients with synonvial chondrosarcoma and found five of 10 had pre-existing chondromatosis [13]. Biazzo et al. found that in a meta-analysis of 67 cases of synovial chondrosarcoma, 81% had evidence of pre-existing synovial chondromatosis [14].

Conventional chondrosarcomas are graded on a scale from 1 to 3. Grade 1 conventional chondrosarcomas typically present as painful, slow-growing masses that elicit reactive thickening of the cortex, while grade 3 tumors present as rapidly growing painful masses that efface the cortex, form soft tissue masses, and metastasize in 70% of cases [12].

The differential diagnosis for the low-grade synovial chondrosarcoma in this case was primary synovial chondromatosis. In our case, the size of the intra-articular bodies differed from that typically seen, with the patient only having a few large intra-articular bodies with thick cartilage caps. In addition, although local recurrences may be common with primary synovial chondromatosis, we believe the rapid local recurrence within two months in our case was somewhat atypical for typical primary synovial chondromatosis.

A prior study by Evans et al. evaluated five cases of synovial chondrosarcomas arising from primary synovial chondromatosis: two cases (40.0%) were grade 1 conventional chondrosarcomas, two cases (40.0%) were grade 2 conventional chondrosarcomas, and one case (20.0%) was a dedifferentiated chondrosarcoma [3]. Histologically, chondrosarcomas are characterized by spotty calcifications, central necrosis, and local invasion into surrounding connective tissue and marrow [10]. Differentiating grade 1 synovial chondrosarcomas from primary synovial chondromatosis is difficult both radiologically and histologically. Histological assessment even on gross resected specimens is subject to sampling error. Grade 1 conventional chondrosarcomas have low cellularity, low pleomorphism, and a low mitotic index, and therefore are difficult to differentiate from synovial chondromatosis.

It is radiographically and histologically difficult to differentiate synovial chondromatosis from low-grade chondrosarcoma. Sperling et al. showed an association between BCL2 expression synovial chondromatosis in four of five patients, and little or no expression in five of six chondrosarcomas [15]. However, this test has not become more utilized in clinical practice.

Treatment for synovial chondrosarcoma is based on grade, and treated with wide, en bloc surgical excision, because chondrosarcomas show limited response to radiotherapy and chemotherapy [12].

In light of these findings, we recommend cautious evaluation of patients with synovial chondromatosis and multiple recurrences especially if the patient has worsening pain or if the disease has any aggressive or atypical imaging features. These patients should be referred to an orthopedic oncologist with expertise in the management of these complex cases.

## Conclusions

Malignant degeneration is a rare but potentially devastating complication of synovial chondromatosis. Practitioners should keep it in mind in patients with worsening or refractory pain, or atypical imaging findings. 
